# Postpartum Depression: Prevalence and Associated Risk Factors Among Women in Sindh, Pakistan

**DOI:** 10.7759/cureus.12216

**Published:** 2020-12-22

**Authors:** Tularam Yadav, Rija Shams, Amir F Khan, Hadiya Azam, Masroor Anwar, Tooba Anwar, Charaghan Siddiqui, Kiran Abbas, Mahnoor Sukaina, Shamas Ghazanfar

**Affiliations:** 1 Internal Medicine, Jinnah Sindh Medical University, Karachi, PAK; 2 Internal Medicine, Jinnah Postgraduate Medical Centre, Karachi, PAK; 3 General Surgery, Lady Reading Hospital MTI, Peshawar, PAK; 4 Internal Medicine, Hayatabad Medical Complex, Peshawar, PAK; 5 Surgery, Lady Reading Hospital MTI, Peshawar, PAK; 6 Internal Medicine, Karachi Medical and Dental College, Karachi, PAK; 7 Internal Medicine, Dow University of Health Sciences, Karachi, PAK

**Keywords:** postpartum depression, breastfeeding woman, formula milk, international classification of diseases - 11 (icd-11), postpartum mental health, peripartum psychosis

## Abstract

Introduction

Postpartum depression (PPD) is defined as the onset of depressive symptoms within six weeks of childbirth. PPD is more common in resource-constrained countries as compared to developed countries. The study aimed to evaluate the factors associated with PPD among women in Sindh, Pakistan.

Methods

A multi-centre, cross-sectional study was conducted at three major tertiary care setups in Sindh, Pakistan. All women presenting to the outpatient department within six weeks of giving live birth were eligible to participate. All women who had stillbirths, abortions, or were treated for a past psychiatric illness or neurological disease were excluded from the study. The Edinburgh postpartum depression scale (EPDS) was used as a screening tool. All socio-demographic factors were documented in a predefined pro forma. The data was analyzed using Statistical Package for Social Sciences (SPSS, Version 26, IBM, Chicago, IL).

Results

According to the Edinburgh postpartum depression scale (EPDS), the incidence of postpartum depression in the current study population was 19.3%. Of these, 12 (3.3%) women had persistently thought about self-harming. Over 100 women did not receive any formal education, constituting the majority of the study population. Formula milk feeding of the newborn was significantly associated with an increased frequency of postpartum depression (p= 0.0001).

Conclusion

The current study highlights the significant burden of postpartum depression in Pakistan. However, the present study failed to find any significant risk factors associated with postpartum depression. Only formula milk feeding was significantly associated with a higher frequency of PPD among study patients.

## Introduction

According to the International Classification Disease (ICD-10), postpartum depression (PPD) is defined as the onset of depressive symptoms within six weeks of childbirth [1,2]. Postpartum depression can manifest in new mothers as feelings of hopelessness, fatigue, unjustified guilt, and shame [3]. Sleep irregularities and reduced appetite in addition to severe mood changes are common symptoms of postpartum depression [4]. 

Globally, postpartum depression can affect up to 15% of mothers annually. PPD is more common in resource-constrained countries as compared to developed countries [5]. The prevalence of postpartum depression in Pakistan ranges from 28-63% [5,6]. Certain factors have been associated with an increased risk of postpartum depression, including a history of psychiatric illness, psychological disturbance during gestation, domestic violence or poor marital relationship, and inadequate social support [7]. Low social status and lack of access to healthcare facilities are also essential factors in determining maternal and fetal outcomes [7,8].

Mothers who suffer from postpartum depression are unable to take care of themselves and provide adequate care to the infant. This results in a non-conducive environment for the personal development of the mothers and their newborn babies. It significantly disrupts infant-mother bonding, leading to child neglect, emotional or physical child abuse‎ [‎9]. Previous studies have suggested that mothers with untreated postpartum depression suffer from weight problems, substance abuse, domestic problems, and breastfeeding issues later in life [9,10]. 

Published data are scarce on the risk factors associated with increased risk of PPD in mothers in Pakistan. Therefore, the current study was conducted to evaluate the factors in predicting PPD among women giving birth at a tertiary care center in Sindh, Pakistan. 

## Materials and methods

A multi-centre cross-sectional study was conducted at three major tertiary care setups in Sindh, Pakistan, which cater to approximately one million patients, annually. The data was collected from the Jinnah Postgraduate Medical Centre (JPMC), Dr Ruth K. M. Pfau, Civil Hospital, and Abbasi Shaheed Hospital. The study was started after obtaining ethical approval from the Institutional Review Board (IRB), reference #JSMU/IRB/2019/-167. A simple random sample technique was used to enrol participants.

All women presenting to the outpatient department within six weeks of giving live birth were eligible to participate. All women who had stillbirths, abortions, or were treated for a past psychiatric illness or neurological disease were excluded from the study. The sample size was calculated via OpenEpi sample size calculator, using a 95% confidence interval (CI), 5.18% margin of error (d), and 48.6% as the prevalence of postpartum depression [10]. A total sample size of 357 was determined. All patients signed informed written consent to participate in the study.

The Edinburgh postpartum depression scale (EPDS) was used as a screening tool. All socio-demographic factors were documented in a predefined pro forma. The EPDS is a self-reported questionnaire with 10 items. It assesses anhedonia, feelings of self-blame, anxiety, fear or panic, inability to cope, and thoughts of self-harm in patients [11]. Due to the participant’s limited English language, the questionnaires were translated into Sindhi, Pashto, and Urdu languages. Native persons critically revised the translated questionnaires to remove any inconsistencies. The questionnaires were then administered to women. Those who could not read or write, the questionnaires were read to them, and their answers were recorded by the authors. 

The data was analyzed using Statistical Package for Social Sciences (SPSS, Version 26, IBM, Chicago, IL). All continuous variables were presented as mean and standard deviation while for all categorical variables, frequency and percentage were used. The dependent variable was the occurrence of postpartum depression, and the socio-demographic variables were tagged as independent variables. Chi-square tests and independent t-tests were used to check for the association between dependent and independent variables. A p-value of less than or equal to 0.05 was considered as significant.

## Results

A total of 357 participants were enrolled in the study. The mean age (standard deviation) was 26.18 (5.53) years and ranged between 14 to 50 years. Three hundred forty-six (96.9%) women were Muslim while only a minority were non-muslims. Over 100 women did not receive any formal education, constituting the majority of the study population (table 1). Out of the 69 women who had postpartum depression, nine (13.1%) had a substance abuse problem. The majority were addicted to areca nuts.

**Table 1:** Sociodemographic Characteristics of Study Participants

Characteristics	N (%)
Religion	
Islam	346 (96.9%)
Hinduism	6 (1.7%)
Christianity	5 (1.4%)
Education Status	
Madrasah	27 (7.6%)
Primary Class	91 (25.5%)
Secondary Class	83 (23.2%)
Higher Secondary Class	26 (7.3%)
Bachelors	29 (8.1%)
No formal education	101 (28.3%)
Occupational Status	
Employed	17 (4.8%)
Unemployed/Homemakers	340 (95.2%)

According to the Edinburgh postpartum depression scale (EPDS), the incidence of postpartum depression in the current study population was 19.3%. Of these, 12 (3.3%) women had persistently thought about self-harming indicating suicidal ideation (Table 2). 

**Table 2:** Study Participants’ Response to the Edinburgh Postpartum Depression Scale

Characteristics	N (%)
I have been able to laugh and see the funny side of things:	
As much as always I could	256 (71.7%)
Not quite as much now	74 ( 20.7%)
Definitely not so much now	15 (4.2%)
Not at all	12 (3.4%)
I have looked forward with enjoyment to things:	
As much as I ever did	284 (79.6%)
Rather less than I used to	51 (14.3%)
Definitely less than I used to	12 (3.4%)
Hardly at all	10 (2.8%)
I have blamed myself unnecessarily when things went wrong:	
Yes, most of the time	91 (25.5%)
Yes, sometimes	42 (11.8%)
Not very often	31 (8.7%)
No, never	193 (54.1%)
I have been anxious or worried for no good reason:	
No, not at all	245 (68.6%)
Hardly ever	18 (5.0%)
Yes, sometimes	63 (17.6%)
Yes, very often	31 (8.7%)
I have felt scared or panicky for no very good reason:	
Yes, quite a lot	21 (5.9%)
Yes, sometimes	57 (16.0%)
No, not much	37 (10.4%)
No, not at all	242 (67.8%)
Things have been getting on top of me:	
Yes, most of the time	34 (9.5%)
Yes, sometimes	69 (19.3%)
No, most of the time	69 (19.3%)
No, I have been coping as usual	185 (51.8%)
I have been so unhappy that I have had difficulty sleeping:	
Yes, most of the time	23 (6.4%)
Yes, quite often	44 (12.3%)
Not very often	43 (12.0%)
Not at all	247 (69.2%)
I have felt sad or miserable:	
Yes, most of the time	10 (2.8%)
Yes, Quite often	23 (6.4%)
Not very often	44 (12.3%)
Not at all	280 (78.4%)
I have been so unhappy that I have been crying:	
Yes, most of the time	23 (6.4%)
Yes, quite often	20 (5.6%)
Only occasionally	34 (9.5%)
No, never	280 (78.4%)
The thought of harming myself has occurred to me:	
Yes, quite often	3 (0.8%)
Yes, sometimes	9 (2.5%)
Hardly ever	11 (3.1%)
No, never	334 (93.6%)

We further stratified the data to assess the different socio-demographic risk factors associated with postpartum depression. We found that the education status of the women, gender of the newborn baby, and type of family did not significantly affect the frequency of postpartum depression among study participants with a p-value of 0.213, 0.595, 0.461, respectively. Formula milk feeding of the newborn was significantly associated with increased frequency of postpartum depression (p = 0.0001) (Table 3).

**Table 3:** The Sociodemographic Factors Associated with Postpartum Depression

Characteristics	EPDS Score		P-value
	Score ≥ 10	Score < 10	
Educational Status			0.213
No formal education	23 (20.3%)	87(30.2%)	
Madrasah or up to class 12^th^	51 (65.2%)	176 (61.1%)	
Bachelors	4(5.8%)	25(8.7%)	
Gender of the Newborn			0.595
Female	29(42%)	104(36.1%)	
Male	40(58%)	183(63.5%)	
Feeding Method			0.0001
Exclusive Breast Feeding	31(44.9%)	191(66.3%)	
Formula Milk Feeding	38(55.1%)	97(33.7%)	
Family Type			0.461
Nuclear	23(33.3%)	83(28.8%)	
Extended	46(66.7%)	205(71.2%)	

## Discussion

We reported an incidence of PPD of 19.3% in our study. Previous studies have shown a PPD prevalence somewhere between 3-69.65%. The varying degrees of prevalence indicate that there are some socio-demographic variables at play here. In developed countries including Singapore, Netherlands, and Switzerland, the prevalence rate of PPD was 3%, 8%, and 11% respectively‎ [12]. However, in less developed and poor constrained countries like Pakistan, India, and Nepal, the incidence of the disease is much higher [3,12-13]. This highlights the significance of social status or resource availability and the risk of PPD in women after delivery. In contrast, the prevalence of PPD was considerably higher in Iran, Chile, South Africa, and Turkey. The reasons for this disparity in different geographic regions is unclear. 

In the current study, we reported a significant association between the educational status of women and the frequency of PPD. PPD was more prevalent in women with poor educational status compared to those with higher education status. In a study by Khan et al., similar conclusions were culminated [14]. Similarly, in a study from Japan, over 90,000 women were evaluated to find an association between education status and risk of developing PPD. The study revealed that higher education status among women was associated with a decreased rate of PPD [15]. The education level of a woman is frequently used to assess the socioeconomic status indirectly. Individuals with lower socioeconomic status have a higher risk of developing a myriad of psychiatric illnesses. The current study reported that PPD was more prevalent in women with lower socioeconomic status highlighting it as an independent risk factor for postpartum depression. 

The present study also found an association between women who were exclusively breastfeeding and a decreased rate of postpartum depression compared to women providing formula milk to their newborns. Previously, a study by Shah et al. reported a significant relationship between the incidence of PPD and exclusive breastfeeding [12]. Ystrom in 2012, revealed that women with postpartum depression or anxiety were a highly vulnerable population. In support of the current findings, these women were likely to cease breastfeeding their newborns after which their symptoms related to depression and anxiety worsened substantially [16].

Our study also found a link between the joint family system and postpartum depression. Women more frequently suffered from PPD if they lived in a joint family system compared to women who resided in a nuclear family system. Naveed and Naz [17] drew the same conclusions about the correlation between the joint family system and increased risk of PPD. Social support is significant for the well-being of new mothers. Essentially, women who did not receive any social support were more prone to suffering from PPD [18]. In a study by Kim et al., it was found that women irrespective of their age had five times the risk of developing postpartum depression if no social support was provided to them after delivery [19]. 

Our study was not without limitations. Firstly, during data collection, the authors and the participants faced language barriers since many of the women belonged to rural areas and could not speak or write the Urdu language fluently. To minimize the language barrier, the authors had the questionnaire translated into several local languages which were then proofread by native persons. However, many women did not have any formal education and were unable to read or write. For such cases, the authors narrated the questionnaires to the participant who then answered accordingly. Secondly, the study was cross-sectional and did not keep a follow-up track of patients. Further studies with longitudinal study designs may help us understand the relationship of these social, psychological, and demographic factors with postpartum depression among women.

## Conclusions

The current study highlights the substantial burden of postpartum depression among women in Sindh, Pakistan. Low education status, family type, and gender of the newborn did not significantly correlate with postpartum depression. However, exclusive formula milk feeding was associated with an increased incidence of PPD among the study population. Interestingly, almost two-fifths of the participants, fed formula milk to their newborns. In short, there is a dire need for attention from the healthcare providers to cater to the mental health needs of pregnant women in our setups.
